# Reasons for admission and variance of body weight at referral in female inpatients with anorexia nervosa in Germany

**DOI:** 10.1186/s13034-021-00427-w

**Published:** 2021-12-22

**Authors:** Triinu Peters, David Kolar, Manuel Föcker, Katharina Bühren, Brigitte Dahmen, Katja Becker, Linda Weber, Christoph U. Correll, Charlotte Jaite, Karin M. Egberts, Marcel Romanos, Stefan Ehrlich, Maria Seidel, Veit Roessner, Christian Fleischhaker, Alexander von Gontard, Freia Hahn, Michael Huss, Michael Kaess, Tanja Legenbauer, Tobias J. Renner, Ulrike M. E. Schulze, Judith Sinzig, Ida Wessing, Gisela Antony, Beate Herpertz-Dahlmann, Gertraud Gradl-Dietsch, Johannes Hebebrand

**Affiliations:** 1 Department of Child and Adolescent Psychiatry, Psychotherapy and Psychosomatics, University Hospital Essen (AöR), University of Duisburg-Essen, Wickenburgstrasse 21, 45147 Essen, Germany; 2 Department of Psychiatry and Psychotherapy, University Hospital, Ludwig-Maximilians-University, Nußbaumstrasse 7, 80336 Munich, Germany; 3 Department of Child and Adolescent Psychiatry Und Psychotherapy, University Medical Center of the Johannes Gutenberg University Mainz, Langenbeckstraße 1, 55131 Mainz, Germany; 4 Department of Child and Adolescent Psychiatry, University Hospital Münster, Schmeddingstraße 50, 48149 Münster, Germany; 5 KBO Heckscher-Klinikum, Deisenhofener Straße 28, 81539 Munich, Germany; 6 Department of Child and Adolescent Psychiatry, Psychosomatics and Psychotherapy, University Hospital, RWTH Aachen, Neuenhofer Weg 21, 52074 Aachen, Germany; 7 Department for Child and Adolescent Psychiatry, Psychosomatics and Psychotherapy, Philipps-University and University Hospital Marburg, Hans-Sachs-Str. 6, 35039 Marburg, Germany; 8 Center for Mind, Brain and Behavior (CMBB), University of Marburg and Justus Liebig University Giessen, Marburg, Germany; 9 Department of Child and Adolescent Psychiatry, Psychosomatics and Psychotherapy, Berlin Institute of Health, corporate member of Freie Universität Berlin, Charité—Universitätsmedizin Berlin, Humboldt-Universität Zu Berlin, Augustenburger Platz 1, 13353 Berlin, Germany; 10 Department of Psychiatry and Molecular Medicine, Zucker School of Medicine at Hofstra/Northwell, Hempstead, NY USA; 11 Department of Psychiatry, The Zucker Hillside Hospital, Northwell Health, Glen Oaks, NY USA; 12 Department of Child and Adolescent Psychiatry, Psychosomatics and Psychotherapy, Centre for Mental Health, University Hospital of Würzburg, Margarete-Höppel-Platz 1, 97080 Würzburg, Germany; 13 Division of Psychological and Social Medicine and Developmental Neurosciences, Faculty of Medicine, TU Dresden, Fetscherstraße 74, 01307 Dresden, Germany; 14 Translational Developmental Neuroscience Section, Department of Child and Adolescent Psychiatry, Eating Disorder Research and Treatment Center, Faculty of Medicine, TU Dresden, Fetscherstraße 74, 01307 Dresden, Germany; 15 Department of Child and Adolescent Psychiatry and Psychotherapy, University Hospital Freiburg, University Freiburg, Hauptstraße 8, 79104 Freiburg, Germany; 16 Department of Child and Adolescent Psychiatry, Saarland University, Kirrberger Straße 1, 66421 Homburg, Germany; 17 Department of Child and Adolescent Psychiatry and Psychotherapy, LVR-Klinik Viersen, Horionstr. 14, 41749 Viersen, Germany; 18 University Hospital of Child and Adolescent Psychiatry and Psychotherapy, University of Bern, Bolligenstrasse 111, Stöckli, 3000 Bern, Switzerland; 19 Clinic of Child and Adolescent Psychiatry, Center for Psychosocial Medicine, University Hospital Heidelberg, Heidelberg, Germany; 20 LWL University Hospital Hamm for Child and Adolescent Psychiatry, Psychotherapy and Psychosomatics, Ruhr University-Bochum, Heithofer Allee 64, 59071 Hamm, Germany; 21 Department of Child and Adolescent Psychiatry, Psychosomatics and Psychotherapy, University of Tübingen, Osianderstraße 14-16, 72076 Tübingen, Germany; 22 Department of Child and Adolescent Psychiatry/Psychotherapy, University Hospital Ulm, University of Ulm, Steinhövelstraße 5, 89075 Ulm, Germany; 23 Department of Child and Adolescent Psychiatry, LVR-Klinik Bonn, Kaiser-Karl-Ring 20, 53111 Bonn, Germany; 24 Institute for Biomagnetism and Biosignalanalysis, University Hospital Münster, Malmedyweg 15, 48149 Münster, Germany; 25 Central Information Office, CIO Marburg GmbH, Struthweg 1, 35112 Fronhausen, Germany

**Keywords:** Anorexia nervosa, Adolescent, Premorbid body weight, Body mass index, Hospital admission, Factor analysis, Treatment seeking behavior

## Abstract

**Background:**

Body mass index (BMI) at hospital admission in patients with anorexia nervosa (AN) represents a prognostic marker for mortality, chronicity and future body weight. The current study focused on the associations between BMI standard deviation score (BMI-SDS) at admission and reasons for seeking inpatient treatment. Further interest was given to the relationship between premorbid weight and weight at admission, as well as the effect of both weight at referral and reasons for admission on treatment outcome.

**Methods:**

Data ascertained in the German Register of Children and Adolescents with AN were analysed to assess the parental and patient overlap for 23 predefined reasons for admission, using factor analyses and regressions models.

**Results:**

Complete parent-patient data sets were available for 360 patients out of 769. The highest consensus rates between parents and patients were obtained for weight and eating behavior related reasons and hyperactivity. Based on factor analysis, four factors emerged. Premorbid BMI-SDS, age and ‘low body weight’ as stated by patients or parents explained almost 40% of the variance of the BMI-SDS at admission.

**Conclusions:**

Results underscore the relevance of age and premorbid BMI for BMI at admission. Only single reasons for admission explained further variance, with ‘low body weight’ having the largest effect. Approximately 40% of the variance of BMI-SDS was explained. For the first time, the effect of premorbid BMI for BMI at admission was robustly demonstrated in a multicenter study. Of the variance in BMI-SDS at discharge, our model could explain 37%, with reasons for admission having a small effect. Further investigation of the reasons for admission would be worthwhile to improve treatment and prognosis.

**Supplementary Information:**

The online version contains supplementary material available at 10.1186/s13034-021-00427-w.

## Introduction

Anorexia nervosa (AN) is an eating disorder with a multifactorial etiology, which can be subsumed into the biopsychosocial model for mental disorders. In routine clinical management, the central therapeutic aim is to enable the patient to gain weight (NICE Guidelines [1]). In a current editorial by Cynthia Bulik [2], the treatment of this eating disorder was judged as inadequate and necessitating a call for action. To improve treatment we obviously need to be in touch with the accounts of patients whose personal stories may focus on “healing, hope and the championing of human spirit in the face of adversity” [3]. At the same time, patients, caregivers and specialists in the field need to generate hypotheses for the treatment of AN, which can subsequently be tested (e.g., [4, 5]).

One of the most profound experiences for a patient in the course of AN is the initiation of inpatient treatment. However, knowledge about presenting complaints and the reasons for seeking inpatient treatment is scant. There is evidence to suggest that coercion plays an important role for the admission process [6]. Adolescent patients experienced stronger external coercion for treatment when they were younger [7]. A low body weight and more severe eating disorder-specific psychopathology were associated with a greater perceived need for hospitalization as assessed by the patients themselves [7]. A qualitative study of the subjective motivations for inpatient care based on application letters of 63 female adult patients with AN [8] revealed three domains of experience, including loss of control, intensity of distress, and ambivalence.

The decision process leading to inpatient child and adolescent psychiatric treatment for AN is complex and includes the evaluation of psychiatric and somatic symptoms as well as the consideration of other patient and family related factors. In addition, the respective health system and national medical practice play an important role. To the best of our knowledge, no study has yet assessed specific somatic and psychiatric reasons for initiation of inpatient treatment for AN in both pediatric patients and their parents. Knowledge of such reasons for admission might lead to a greater understanding of the factors driving patients and their parents to seek treatment and might facilitate engagement in treatment programs. If subgroups of patients with AN can be identified at the time of admission, patients could potentially receive a more individualized treatment. Accordingly, this study aimed to assess the endorsed frequencies of 23 predefined reasons for seeking inpatient treatment separately in patients and their parents. We hypothesize that the endorsed reasons only partially overlap between patients and parents due to differences in symptom perception and assessment of severity and urgency for treatment. We assume that clusters exist for reasons for seeking hospital treatment, thus allowing identification of symptom-related subgroups of patients. We finally hypothesize that the reasons for admission explain part of the variance in BMI at initiation of inpatient treatment. From a medical perspective, knowledge of factors explaining variance of BMI upon admission for inpatient treatment is important. Thus, body mass index (BMI; kg/m^2^) at admission for inpatient treatment for anorexia nervosa (AN) is used to grade the severity of this eating disorder [9]. BMI at admission represents a prognostic marker for mortality, chronicity and future body weight [10–12]. BMI at admission for inpatient treatment of pediatric patients with AN has recently been shown in a large register-based data set to age-dependently increase up to age 15 years with a subsequent plateau [13]. Apart from age, BMI at admission also depends on premorbid body weight [14–16] with a higher premorbid BMI predicting a higher BMI at admission.

## Methods

### Participants and assessment measures

Data of patients of the multi-center German Register of Children and Adolescents with AN [17–19] was used for the current analysis. Inpatients with a DSM-5 diagnosis of AN or Other Specified Feeding or Eating Disorder with an AN-like symptomatology (two out of the three DSM-5 criteria fulfilled; subsequently referred to as non-typical AN) were enrolled by the 16 participating child and adolescent psychiatric hospitals between August 2014 and May 2020. All patients admitted to one of the participating centers were eligible for entry of their data into the register provided that both patient and caregivers agreed. At the time of the analysis the multi-center register included data of 769 patients. As a result of missing or unavailable data (a common limitation in register based studies; see [20] available sample sizes based on different variables vary.

The ethics committees of all participating universities (n  = 14) and local research centers (n  = 2) approved the register study. A written informed assent/consent of both patient and at least one legal guardian was available.

For the current study, patients were included with complete parent-patient dyad data for: (i) DSM-5 diagnosis of AN or non-typical AN (see above), (ii) age upon admission in years and months, not older than 18 years, (iii) height and weight upon admission measured in underwear and without shoes using calibrated hospital scales and stadiometers, (iv) date upon admission, (v) recalled premorbid weight defined as the body weight in kilograms prior to initial weight loss, (vi) recalled month and year, at which recalled premorbid weight last applied, (vii) up to three reasons for seeking inpatient treatment provided by the patient and/or one or both parents. As data from only 22 male patients had been included in the register, this analysis was only carried out with female patients. Data on sex was missing for 53 patients, who were also excluded.

The DSM-5 eating disorder was diagnosed by the treating clinician at each of the local centers. DSM-5 criteria for AN were provided in the register data entry to increase reliability of the diagnoses; a BMI up to the 10th sex and age-adjusted percentile was defined as being still compatible with the DSM-5 A (weight) criterion. For 73% of the patients this was the first inpatient treatment, 27% of the patients had been hospitalized at least once before.

BMI at admission was calculated by dividing measured body weight by the square of measured height (kg/m^2^) upon admission. Premorbid BMI was estimated based on recalled body weight prior to initial weight loss and measured height upon admission (for details see [14, 15]). On the basis of nationally representative German reference data for children (KiGGS) [21], individual BMI-values were transformed into BMI-Standard Deviation Scores (BMI-SDS) and BMI-percentiles using the method suggested by Cole and Green [22]. The method has been adapted for the calculation of BMI-SDS [23]. The BMI-SDS approximates the deviation of an individual BMI from the median of the reference group expressed in units of the standard deviation.

Patients and parents were asked separately to provide up to three major reasons for seeking inpatient treatment according to a list of predefined items. Participants were probed with the following question: “We are attempting to understand the major reasons for your seeking inpatient treatment. We will read a list of such reasons to you and ask you to provide us with the most important reasons. You can choose up to three items from the list. The items were generated by four experienced board-certified child and adolescent psychiatrists (KBü, MF, JH, BH-D) with an expertise in eating disorders based on clinical expertise; as such, the predefinition of these reasons was based on a medical perspective of AN. Each of the 23 items was recorded dichotomously (positive/negative).

## Statistical analyses

To obtain a descriptive overview of the reasons given for inpatient treatment we calculated absolute and relative frequencies of each item (reason). To investigate the probability that both patients and parents endorsed the same reason, we calculated the point estimate for the probability according to the binomial distribution (p  =  k/n; k representing the number of times the respective reason was endorsed by both patients and parents; n representing the number of times a reason was affirmed by at least one of the two groups) for each of the 23 reasons. 95% confidence intervals (95% CI) were calculated with the Clopper-Pearson interval based on the cumulative probabilities of the binominal distribution (inverse beta-distribution).

We additionally performed a factor analysis using polychoric correlations to identify clusters of items separately for patients and parents. Prior to the performance of the factor analysis, we calculated a correlation matrix to test for common variance based on the Kaiser–Meyer–Olkin (KMO) measure of sample adequacy [24]. The KMO statistic varies between 0 and 1, values  < 0.5 are regarded as inacceptable, values between 0.6 and 0.7 as mediocre, values between 0.7 and 0.8 as good, values between 0.8 and 0.9 as great and values  > 0.9 as superb [25].

In order to achieve a KMO  > 0.5 in the correlation matrix, we considered correlation coefficients between items. To test the overall correlations objectively, we used Barlett’s test, which tests whether the correlation matrix is an identity matrix. If a significant result can be obtained, the items cannot be considered as independent of each other. To test for multicollinearity, we tested the determinant of the correlation matrix, which should be  > 0.00001 to assume that multicollinearity does not apply. Principal component analyses (PCA) were conducted with varimax rotation.

We calculated Pearson’s correlation coefficients between premorbid BMI-SDS, BMI-SDS and age upon admission. To explain the variance of BMI-SDS at admission we calculated regression models with age, premorbid BMI-SDS, duration of illness (log-transformed) and initial admission vs.  ≥ 1 pretreatment episode as independent variables. Because 25 independent variables (predictors) were too many in a regression model, single items (reasons for admission) were introduced into the resultant regression model including age and premorbid BMI-SDS with models calculated separately for patients and parents. All items which achieved nominal significance were incorporated into the final regression model and subsequently corrected for multiple testing (Bonferroni-Holm correction [26]). The same approach was taken in the post-hoc analysis to investigate the effect of the reasons for admission on BMI-SDS at discharge. Correlation coefficients between premorbid BMI-SDS, BMI-SDS at admission, age at admission, duration of treatment, weight gain per week and BMI-SDS at discharge were calculated. To explain the variance of BMI-SDS at discharge we calculated regression model with premorbid BMI-SDS, age at admission, BMI-SDS at admission, duration of treatment and weight gain per treatment weak. Single items (reasons for admission) were introduced into this regression model. For descriptive post-hoc analyses independent T tests and χ^2^-tests were performed. Exact two-sided significances were calculated, the alpha level was set to 0.05. Analyses were performed using IBM^®^ SPSS^®^ Statistics 25.0.0.1 for Windows and R (R Core Team (2018), a language and environment for statistical computing. R Foundation for Statistical Computing, Vienna, Austria, https://www.R-project.org/).

## Results

The descriptive comparison of the complete parent-patient dyads of 360 patients with reasons for admission, and the data set used for factor analysis in patients (n  =  471) revealed only minor differences in the respective variables (Table 1). Most patients (81%; complete parent-patient dyad data) had the restricting type of AN. Fourteen percent of the patients were classified as non-typical AN. The mean premorbid and BMI-SDS at admission were − 0.36 (SD  = 0.89) and − 2.96 (SD  = 1.1; based on n  = 369 patients).

**Table 1:** Sample characteristics

	Complete parent-patient dyad data set (n = 360)		Data set used for factor analysis based on patients’ reasons given for inpatient treatment (n = 471)		
	Mean	SD	Data available for n cases	Mean	SD
Age at admission (years)	15.16	1.59	471	15.10	1.78
BMI at admission (kg/m^2^)	15.26	1.50	471	15.23	1.51
BMI-SDS at admission	− 2.96	1.18	471	− 2.96	1.21
Premorbid BMI (kg/m^2^)	19.68	2.76	424	19.69	2.85
Premorbid BMI-SDS	− 0.36	0.89	412	− 0.36	0.91
Illness duration (weeks)	51.11	45.30	448	52.89	54.53

	Absolute frequency	Relative frequency	Data available for n cases	Absolute frequency	Relative frequency
DSM-5 diagnoses			471		
AN restricting type	292	0.81		377	0.80
AN binge-eating/purging type	20	0.06		23	0.05
Non-typical AN^a^	48	0.13		71	0.15

*SD* standard deviation; *BMI* body mass index; *BMI-SDS* BMI standard deviation score

^a^Non-typical AN refers to inpatients with an AN-like clinical symptomatology, in whom any combination of two of the three DSM-5 criteria applied upon admission

Four hundred and twenty patients and at least one parent mentioned up to three reasons for admission, thus allowing their comparison. Symptoms of AN related to body weight (‘low body weight’, ‘rapid weight loss’) and the item ‘reduced food intake’ were commonly endorsed by both patients and parents (joint affirmation in  > 40% of cases; see Table 2). A high degree of joint affirmation was also apparent for ‘hyperactivity’, which was however endorsed by only 11 patients and 13 parents. ‘Depressed mood’ and ‘recommendation of a physician or therapist’ were also frequently mentioned as one of up to three major reasons for seeking inpatient treatment. Somatic reasons for treatment seeking were infrequently endorsed. The 95% confidence intervals for the point estimates were large for items that were named infrequently.

**Table 2:** Reasons for hospital admission by 420 patients and their parents and the probability of an overlap

Item	Frequencies [n (%)] parents (n = 420)	Frequencies [n (%)] patients (n = 420)	Frequency overlap parents/patient k	Point estimate for probability (p) (%)	95% confidence interval (%)
Low body weight	273 (65.0)	179 (42.6)	150	49.7	44.0–55.5
Rapid weight loss	193 (46.0)	117 (27.9)	93	42.9	36.2–49.7
Reduced food intake	218 (51.9)	153 (36.4)	111	42.7	36.6–49.0
Complete cessation of food and/or fluid intake	21 (5.0)	19 (4.5)	7	21.2	9.0–38.9
Vomiting (self-induced)	17 (4.0)	14 (3.3)	8	34.8	16.4–57.3
Hyperactivity	13 (3.1)	11 (2.6)	7	41.2	18.4–67.1
Depressed mood	108 (25.7)	103 (24.5)	54	34.4	27.0–42.4
Suicidal ideation	11 (2.6)	20 (4.8)	7	29.2	12.6–51.1
Social withdrawal	45 (10.7)	53 (12.6)	15	18.1	10.5–28.0
Compulsions	21 (5.0)	20 (4.8)	9	28.1	13.7–46.7
Other mental problems	6 (1.4)	9 (2.1)	2	15.4	2.0–45.4
Exhaustion	23 (5.5)	59 (14.0)	9	12.3	6.0–22.1
Dizziness	10 (2.4)	20 (4.8)	6	25.0	9.0–46.7
Low heart rate	18 (4.3)	12 (2.9)	6	25.0	9.0–46.7
Low blood pressure	5 (1.2)	3 (0.7)	1	14.3	0.4–57.9
Abnormal electrocardiogram	8 (1.9)	4 (1.0)	2	20.0	2.0–55.6
Abnormal blood laboratory values	3 (0.7)	5 (1.2)	1	14.3	0.4–57.9
Other somatic symptoms	4 (1.0)	5 (1.2)	0	0	nc-33.6
Increase in school absenteeism	4 (1.0)	6 (1.4)	0	0	nc-30.8
Family conflicts	60 (14.3)	51 (12.1)	24	27.6	18.5–38.2
Recommendation of physician or therapist	98 (23.3)	121 (28.8)	55	33.5	26.4–41.3
No reason for admission	1 (0.2)	27 (6.4)	0	0	nc-12.3
Other reasons	29 (6.9)	40 (9.5)	14	25.5	14.7–39.0

*k* both parents and patients affirmed a specific reason; *p* the probability of an overlap in affirmed reasons; *nc* not computable

### Factor analyses

Factor analyses were conducted separately for patients and parents. Complete data sets including patient *or* parental reasons for admission were available for 471 and 470 patients, respectively. The polychoric correlations between items were mostly low (r  < 0.3; Additional file 1: Tables S1, S2). The highest correlation coefficient was 0.59 (between ‘low heart rate’ and ‘recommendation of physician or therapist’) for patients and 0.60 (between ‘abnormal blood laboratory values’ and ‘abnormal electrocardiogram’) for parents.

We achieved a matrix with a KMO  > 0.5 based on ten items each for patients (KMO  = 0.63; Table 3a) and parents (KMO  = 0.62, Table 3b). The KMO values are equivalent to a mediocre degree of common variance. For patients and parents, the common variance matrices based on ten items each provided KMO-values of 0.63 and 0.62, which represent a mediocre degree of common variance (Table 3). Bartlett’s tests for sphericity were significant [patients: χ^2^(45)  =  11,474.43, P  < 0.0001]; parents: [χ^2^(55)  = 11,029.79, P  < 0.0001], indicating that correlations between items were sufficiently large for PCA. The determinants of the correlation matrices were 2.0 × 10^–11^ in patients and 4.9 × 10^–11^ in parents, thus suggesting that collinearity cannot be excluded for both respondents. The initial analysis in patients showed that four components had eigenvalues  ≥ 1. In combination, these components explained 74% of the variance. Similarly, in parents four components had eigenvalues  ≥ 1, which in combination explained 69% of the variance (see scree plots in Additional file 1: Figs. S1, S2). Model fit was 0.88 (0.95 indicates a good fit) for patient data and 0.82 for parent data. Histograms of model residuals are shown in Additional file 1: Figs. S3, S4.

**Table 3:** Summary of exploratory factor analysis based on reasons for admission reported by 471 patients and 470 parents

Item	Factor 1^a^	Factor 2	Factor 3	Factor 4
(a) Patients				
Exhaustion	**0.74**			
Dizziness	**0.74**	0.41		
Low body weight	**−** **0.73**		− 0.32	
Abnormal blood laboratory values		**0.89**		
Increase in school absenteeism	0.54	**0.69**		
Reduced food intake		**−** **0.56**	− 0.32	0.38
Complete cessation of food and/or fluid intake			**0.83**	
Suicidal ideation			**0.83**	
Rapid weight loss			− 0.34	**0.81**
Low blood pressure		0.44		**0.77**
(b) Parents				
Abnormal blood laboratory values	**0.86**			
Abnormal electrocardiogram	**0.75**			
Low blood pressure	**0.66**			
Complete cessation of food and/or fluid intake		**0.77**		
Reduced food intake	− 0.4	**−** **0.76**		
Compulsions		**0.71**	0.47	
Recommendation of physician or therapist			**−** **0.96**	
Hyperactivity	0.42		**0.47**	
Low body weight				**−** **0.81**
Suicidal ideation				**0.79**

^a^Standardized factor loadings after rotation  > 0.3 are shown

‘Low body weight’, ‘low blood pressure’, ‘abnormal blood laboratory values’, ‘reduced food intake’, ‘complete cessation of food and/or fluid intake’, ‘suicidal ideation’ could be combined into factors in the analyses based on both patient and parental data (Table 3).

The first factor in patients summarized somatic problems like ‘exhaustion’, ‘dizziness’ and ‘low body weight’. ‘Low body weight’ had a negative correlation to this factor. The second factor combined problems such as ‘abnormal blood values’, ‘increased absenteeism from school’ and ‘reduced food intake’, with ‘reduced food intake’ having a negative loading to this factor. The third factor summarized ‘complete cessation of food and/or fluid intake’ and ‘suicidal ideation’. The fourth factor again consisted of somatic problems: ‘rapid weight loss’ combined with ‘low blood pressure’.

Similar to patient data, the first factor, calculated on the basis of parent data, summarized somatic problems: ‘abnormal blood laboratory values’, ‘abnormal electrocardiogram’ and ‘low blood pressure’. The second factor included psychiatric problems such as ‘compulsions’ associated with ‘complete cessation of food and/or fluid intake’ and ‘reduced food intake’, with ‘reduced food intake’ again having a negative correlation with this factor. The third factor combined two items: ‘recommendation of physician or therapist’ and ‘hyperactivity’, these items having opposite correlations to this factor. The fourth factor consisted of two items, ‘suicidal ideation’ and ‘low body weight’, whereby the last one again had a negative loading to this factor.

### Correlation and regression analyses

Pearson correlations between premorbid BMI-SDS and BMI-SDS at admission and between BMI-SDS at admission and age were 0.47 (P  = 4.7 × 10^–21^) and − 0.34 (P  = 2.4 × 10^–11^), respectively (n  = 360).

The baseline regression analysis (model Ia) sought to explain BMI-SDS at admission by premorbid BMI-SDS, age at admission, illness duration (ln) and initial treatment vs.  > 1 pretreatment episode. This model explained 34% of the variance of BMI-SDS at admission. Premorbid BMI-SDS had the largest effect size. Despite the fact that BMI-SDS represents an age-adjusted assessment of BMI in relationship to a population based reference sample, age had a negative effect in the model. Because duration of illness and initial vs.  ≥ 1 pretreatment episode were not significant predictors, we omitted them in the final model (model Ib). This model Ib revealed a similar result (R^2^  = 0.33) (Table 4).

**Table 4:** Results of regression models Ia and Ib with BMI-SDS at admission as outcome (n  = 360)

	B	SE	Beta	Sig.^a^	B	SE	Beta	Sig.^a^
Intercept	1.077	0.501		**0.032**	1.020	0.487		**0.037**
Premorbid BMI-SDS	0.626	0.058	0.473	**1.002 × 10**^**–23**^	0.611	0.057	0.464	**2.05** × **10**^**–23**^
Age at admission	− 0.222	0.035	− 0.300	**1.025 × 10**^**–9**^	− 0.248	0.032	− 0.336	**9.26** × **10**^**–14**^
Illness duration (ln)	− 0.117	0.076	− 0.079	0.127				
Pre-treatments (no/yes)	− 0.058	0.128	− 0.022	0.652				
	Model Ia: R^2^ = 0.34				Model Ib: R^2^ = 0.33			

*B* unstandardized regression coefficients; *SE* standard error of unstandardized regression coefficient; *Beta* standardized coefficients; *Sig* significance; *R*^*2*^ explained variance

^a^Nominal significant P values are bolded

To assess the influence of the dichotomous items (reasons for admission), we extended the baseline regression model Ib based on premorbid BMI-SDS and age at admission to include each of the 23 items for patients and parents, respectively (Additional file 1: Table S3a, b available online). The slightly different regression coefficients for age and premorbid BMI-SDS compared with model Ib are not shown. The change in R^2^ (ΔR^2^) indicates the improvement of the respective model compared to model Ib. Nominally significant items/reasons (P  < 0.05) are highlighted.

In patients, only three items achieved nominal significance (‘low body weight’, ‘depressed mood’ and ‘other reasons’), of which ‘low body weight’ had the largest (negative; due to the dichotomous coding of the reasons (0  =  reason not endorsed, 1  =  reason endorsed) effect (Additional file 1: Table S3a, available online). Upon inclusion of these three items in the final model, 39% of the variance of BMI-SDS at admission were explained (Table 5). All predictors in the final model survived the Bonferroni-Holm correction.

**Table 5:** Results of regression analyses with BMI-SDS as outcome (n  = 360)

Predictors^a^	B	SE	Beta	t	Sig.^b^
(a) Patients (R^2^ = 0.39)					
Intercept	1.14	0.47		2.42	**0.016**
Premorbid BMI-SDS	0.57	0.06	0.43	10.27	**7.63** × **10**^**–22**^
Age at admission	− 0.25	0.03	− 0.34	− 8.19	**4.85** × **10**^**–15**^
Low body weight	− 0.38	0.10	− 0.16	− 3.74	**0.00022**
Depressed mood	0.28	0.11	0.10	2.47	**0.014**
Other reasons	0.41	0.17	0.10	2.37	**0.018**
(b) Parents (R^2^ = 0.42)					
Intercept	1.01	0.47		2.15	0.03
Premorbid BMI-SDS	0.55	0.05	0.42	10.19	**1.56** × **10**^**–21**^
Age at admission	− 0.23	0.03	− 0.32	− 7.55	**3.86** × **10**^**–13**^
Low body weight	− 0.46	0.11	− 0.19	− 4.28	**2.47** × **10**^**–05**^
Vomiting (self-induced)	0.56	0.25	0.10	2.29	0.023
Depressed mood	0.16	0.12	0.06	1.41	0.160
Suicidal ideation	0.58	0.29	0.08	1.95	0.052
Social withdrawal	0.34	0.16	0.09	2.19	0.029
Abnormal electrocardiogram	− 0.77	0.38	− 0.08	− 2.04	0.043
Recommendation of physician or therapist	− 0.10	0.12	− 0.04	− 0.88	0.382

*B* unstandardized regression coefficients; *SE* standard error of unstandardized regression coefficient; *Beta* standardized coefficients; *t* t statistic; *Sig* significance; *R*^*2*^ explained variance

^a^All items rendering nominal P values  < 0.05 based on reasons for inpatient treatment given by patients and parents

^b^Predictors that survive the Bonferroni-Holm correction are highlighted (6 tests in model for patients, 10 tests in model for parents)

Seven reasons affirmed by parents achieved nominal significance (Additional file 1: Table S3b, available online) with ‘low body weight’ again having the strongest effect. The final model including these seven items explained 42% of the variance of BMI-SDS at admission (Table 5). Only ‘low body weight’ survived the correction for multiple testing. The explained variance based on the inclusion of ‘low body weight’ as the only reason surviving correction for multiple testing was 38%.

### Post-hoc analyses

For factor 1 in patients, we investigated the differences in BMI-SDS between patients who reported “fatigue” and/or “dizziness” and those who did not. Both BMI-SDS at admission (‘exhaustion’ yes/no: − 2.90/− 2.97, P  = 0.673; ‘dizziness’ yes/no: − 2.68/− 2.97, P  = 0.278) and premorbid BMI-SDS (‘exhaustion’ yes/no: − 0.30/− 0.37, P  =  0.533; ‘dizziness’ yes/no: − 0.09/− 0.37, P  = 0.198) were non-significantly higher in patients endorsing the respective reasons.

Based on the results obtained for “low body weight” in the regression analysis, we analysed to what extent this item entails a lower body weight. If “low body weight” was endorsed, BMI-SDS at admission was substantially lower than in patients, who themselves or whose parents did not affirm this reason (admission BMI-SDS − 3.40 versus − 2.64 and − 3.19 versus − 2.42 in patient and parental data, respectively (P  < 1 × 10^–11^ for both comparisons).

A descriptive overview of measurements at discharge is presented in Additional file 1: Table S4. After the average treatment duration of 17.21 weeks (SD  = 8.47), patients achieved a mean BMI of 18.26 (SD  = 1.43), corresponding to a mean BMI-SDS of − 1.15 (SD  = 0.77). The correlation coefficients between different measurements before the onset of AN, at admission and at discharge are shown in Additional file 1: Table S5. The basic model to explain the variance of BMI-SDS at discharge captured the predictors premorbid BMI-SDS, BMI-SDS at admission, age at admission, duration of treatment and weight gain per treatment weak. This model explained 34% of variance of BMI-SDS at discharge (Additional file 1: Table S6). Of a total of 23 reasons asked of parents, only 'other mental problems’ and ‘recommendation of physician or therapist’ had a significant effect on BMI-SDS at discharge. The final model explained 37% of the variance in BMI-SDS at discharge (Additional file 1: Table S6a, b). Patients’ self-reported reasons for admission had no significant association with BMI-SDS at discharge (data not shown).

## Discussion

This study explained almost 40% of the variance of the clinically important BMI-SDS at admission for inpatient treatment in a large sample of pediatric patients with AN. The explained variance based on premorbid BMI-SDS, age at admission, and single reasons for admission as delineated by patients or their parents exceeded the 27% explained in our previous study based on premorbid BMI age percentile and age at admission of 161 patients [15]. Hence, our results indicate that the explained variance can be increased by including reasons for admission in regression models. In addition, we describe for the first time such reasons based on the accounts of both patients and parents, thereby enabling both comparisons and factor analyses.

The current study again underscores the relevance of premorbid BMI for BMI at admission. For the first time, this effect was robustly demonstrated in a multicenter study based on a large number of patients. The mean premorbid BMI-SDS of our patients was − 0.36 (SD  = 0.89) which suggests a deviation towards a lower-than-average premorbid BMI. Indeed, some investigators have observed a general predisposition to underweight in patients with AN prior to onset of the eating disorder [27, 28]. In addition, the most recent genome wide association analysis for AN detected a negative genetic correlation between this eating disorder and BMI [29]. On the other hand, evidence of a more frequent occurrence of premorbid overweight has also been reported in patients with AN [30]. Our negative BMI-SDS may in part reflect the influence of overestimated height (entailing a too low estimate for premorbid BMI-SDS) due to the use of measured height at referral. It should also be pointed out, that—while not assessed in the current study—patients with AN tend to stem from families with an above average educational background [31], which is associated with a lower mean BMI [32–34]. Finally, our results only provide a glimpse at premorbid body weight at the time point of recalled initial weight loss; accordingly, an assessment of childhood body weight is not possible.

In both the current and our previous study [15] age at admission was a significant predictor of BMI at admission. Accordingly, the use of sex- and age-adjusted premorbid BMI (current study: BMI-SDS; Föcker et al. [15]: BMI-percentile) does not entail the absence of an effect of age. Using an overlapping data set we recently showed that both BMI and BMI-SDS at hospital admission are age-dependent. Mean BMI at admission age-dependently increased only in those patients aged  < 15 years upon admission; in contrast, for BMI-SDS an age dependency was observed only in patients aged  ≥ 15 years upon admission [13]. We had hypothesized that these age dependencies are related to changes in absolute and percent body fat mass during puberty.

Interestingly and potentially in contrast to clinical expectations, duration of illness in this study again was not a significant predictor of BMI at admission (Table 4). Since the results are based on a large data set, we conclude that illness duration does not have a clinically relevant, independent effect on BMI at admission. A recent meta-analysis found no effect of duration of illness on treatment outcome [35].

Both patients and parents were asked to provide up to three reasons for admission for inpatient treatment. Clearly, the reasons differed (Table 2). Parents much more frequently endorsed reasons related to both body weight (‘low body weight’, ‘rapid weight loss’) and ‘reduced food intake’. ‘Exhaustion’, ‘dizziness’ and ‘suicidal ideation’ were mentioned twice as frequently by patients compared to their parents. Somatic symptoms (‘low blood pressure’, ‘low heart rate’, ‘abnormal ECG’ and ‘abnormal laboratory values’) were mentioned infrequently only. Whereas the parent(s) of only one patient stated that there was no reason for admission, 29 patients endorsed the respective item, potentially suggesting an oppositional component and/or depressed mood and/or reduced cognition upon admission. In conclusion and not unexpectedly, it appears that weight related items and reduced food intake are more of a concern to parents; patients seem to feel less restricted by these symptoms potentially due to their ego-syntonic nature in anorexia nervosa. It is, however, interesting, that patients admitted to being exhausted, having dizzy spells and suicidal ideation. These findings are in line with a study examining perceptions among patients with eating disorders about their need for hospitalization and the coerciveness of the admission process. Adolescent patients reported more perceived coercion than did adult patients and were more likely to disagree that they needed hospitalization [6].

The highest consensus rates (point estimate  > 40%) between parents and patients were obtained for clinically readily discernible features of AN (‘low body weight’, ‘rapid weight loss’, ‘reduced food intake’ and ‘hyperactivity’). ‘Vomiting’ (34.8%) and ‘depressed mood’ (34.8%) also revealed high point estimates. ‘Family conflicts’ were endorsed by 60 parents and 50 patients (point estimate 25.5%). The fact that 29 parents and 40 patients endorsed ‘other reasons’ with a point estimate of 25.5% indicates that as expected our list of 23 reasons did not cover all applicable reasons. In case of endorsement of other reasons by patients, these were entered as free text; examples include “hope for a normal life”, “loss of quality of life”, “circling of thoughts”, ‘parental wish’, ‘judge’s order’ etc. Free text explanations given by parents included ‘sleeping problems’, ‘panic attacks’, ‘constipation’, ‘change of character’, ‘concern about possible physical harm’, ‘daily discussions about eating’, etc. Some of these reasons, such as ‘constipation’ or ‘panic attacks’, could have been attributed to’mental problems’ or ‘other somatic symptoms’ upon data entry. In future research, ‘other reasons’ could be differentiated into (a) rare symptoms and (b) external reasons such as ‘judge’s order’, ‘parental wish’ or (c) motivation to overcome AN such as ‘hope for a normal life’ or ‘renewed participation in competitive sports’.

The factor analyses revealed four albeit different factors in both patients and parents based on ten items each, of which five overlapped between patients and parents (‘low body weight’, ‘reduced food intake’, ‘complete cessation of food and/or fluid intake’, ‘suicidal ideation’, ‘abnormal blood laboratory values’). ‘Depressed mood’ as a reason for admission was mentioned relatively frequently (patients: n  = 103; parents n  = 108), but due to weak correlations to other items, ‘depressed mood’ could not be included in the factor analysis. Despite the fact that somatic reasons for admission were endorsed rather infrequently, they figured prominently in the factors. Interestingly, according to parents, three somatic items (‘abnormal blood laboratory values’, ‘abnormal electrocardiogram’, ‘low blood pressure’) loaded positively on a single factor (see Factor 1 in Table 3, parents); in addition, ‘low blood pressure’ loaded positively on Factor 4 together with ‘rapid weight loss’ (see Table 3, patients). Similarly, ‘exhaustion’ and ‘dizziness’ reported by patients loaded positively on Factor 1 (see Table 3, patients), whereas a ‘low body weight’ loaded negatively on the same factor. Thus, patients who cited ‘exhaustion’ and/or ‘dizziness’ as one of the three most important reasons for admission were less likely to mention the reason ‘low body weight’. A post-hoc analysis revealed that these patients had a non-significantly higher BMI-SDS at admission and premorbid BMI-SDS than other patients. Possibly, the negative correlation of low body weight with factor 1 can in part be explained by the fact that for these patients reporting ‘exhaustion’ and/or ‘dizziness’, a ‘low body weight’ was not relevant enough as one of the three most important reasons. For parents, objectively measurable somatic reasons (‘abnormal blood laboratory values’, ‘low blood pressure’, and ‘abnormal electrocardiogram’) loaded on factor 1, but in patients perceptible but not objectively measurable somatic problems such as ‘exhaustion’ and ‘dizziness’ loaded on one factor. In parents, ‘low body weight’ loaded negatively on Factor 4, which additionally encompassed ‘suicidal ideation’. In patients, ‘suicidal ideation’ and ‘complete cessation of food and/or fluid intake’ both loaded on the same factor (see Factor 4 in Table 3, patients). In both patients and parents data ‘low body weight’ loaded negatively on the respective factors. Overall, our results point to specific reasons, which warrant further investigations in an attempt to come up with specific clusters deemed relevant upon hospital admission. These clusters, which possibly also differentiate subgroups of patients with AN, may also be of prognostic and therapeutic importance. Such subgroups may differ, for example, in terms of patients' motivation to change (see Vansteenkiste et al.) [36]. We refrained from using the results of the factor analyses for data reduction in our regression models, because only ten items could be included in factor analyses in both patients and parents. In addition, some key measures (model fit, residuals, collinearity) of the models were not deemed as sufficiently good.

The major goal of our study was to assess if the inclusion of specific reasons for seeking inpatient treatment increases the explained variance of BMI at admission beyond that obtained upon use of the variables premorbid BMI-SDS and age. In particular, the endorsement of a ‘low body weight’ by patients and/or parents proved to be a highly significant predictor that explained between 3.5% (patients) and 5% (parents) of the variance of BMI at admission. At first glance, this may appear tautological in that it appears obvious that a low body weight at admission should predict a low BMI. However, patients or parents endorsed this particular reason out of 23 possible ones without a solid knowledge of the distribution of BMI at admission of patients with AN, thus indicating a valid perception of the degree of underweight for those patients/parents who view a low body weight as a major reason for admission. As such, if patients or parents endorse a low body weight as a major reason for admission, they seemingly realize that body weight is low indeed.

Apart from a low body weight only single items achieved nominal significance in the regression models including a single reason in addition to premorbid BMI-SDS and age at admission (Additional file 1: Table S3a, b available online). The effect of each of these items on explained variance was small in both patients and parents. Upon consideration of nominally significant items, the final regression models predicted around 40% of the variance of BMI at admission, which as pointed out above presumably represents an underestimation. We consider this proportion of explained variance as high upon consideration of the multiple factors influencing admission for inpatient treatment including for example a consensus between patient and parents to seek inpatient treatment, medical and psychological advice during outpatient contacts prior to admission, and availability of a hospital bed.

We conducted post-hoc analyses to examine the associations between AN symptoms (recorded as reasons for admission) and treatment outcome. We measured treatment outcome by weight at discharge (BMI-SDS). Indeed, weight is the most important criterion for the assessment of treatment success during inpatient treatment. Reasons for admission given by parents contributed little to the explained variance of BMI-SDS at discharge: when the reason ‘other mental problems’ was given, BMI-SDS at discharge was lower and when ‘recommendation of physician of therapist’ was given, BMI-SDS was higher. None of the reasons given by patients themselves were related to weight at discharge. It is of interest to point out that premorbid BMI-SDS remained a minor but nevertheless significant predictor of BMI-SDS at discharge despite the inclusion of BMI-SDS at admission in the model.

The limitations of our study merit consideration. Due to the use of data from a national register which involves 16 university and non-university hospitals, the results of this study are subject to the typical strengths and limitations of register studies [20]. The comparability of our results may be limited due to variation among different mental health care systems pertaining to treatment settings and admission policies. However, a systematic review comparing available evidence-based clinical treatment guidelines for specific eating disorders from eight different countries indicated that most guidelines provided criteria for hospitalization [37]. The guidelines require consistently that the decision for inpatient treatment for patients with AN should be made on an individual basis. This should include identifying multiple factors such as a high risk for medical complications as determined by the weight status, behavioural factors, vital signs, psychiatric comorbidity (e.g., suicide risk) or environmental factors (e.g., family support) [37]. In our study, the most commonly endorsed reasons for seeking inpatient treatment by both patients and parents were symptoms of AN directly or indirectly related to body weight (‘low body weight’, ‘rapid weight loss’, ‘reduced food intake’). ‘Depressed mood’ was also frequently mentioned as one of up to three major reasons for seeking inpatient treatment. We therefore assume that our results should be comparable to other inpatient treatment facilities for AN.

It should be pointed out that our study did not aim to address the medical indications for inpatient treatment, its efficacy nor its side effects. The results of our study need to be viewed from the perspective of the German health care system, in which inpatient treatment is in comparison to other countries a more common form of treatment for children and adolescents with AN. In Germany, admission to inpatient treatment is usually based on a referral from the treating physician or psychotherapist. Accordingly, future studies could also attempt to assess the reasons for inpatient treatment from the perspective of the referring physician or psychotherapist. We are aware of only one study that investigated the information content of referral letters for psychotherapeutic treatment of children and adolescents in the Netherlands. Among the total of n  = 723 patients, there were only n  = 27 patients with feeding and eating disorders [38]. Because of the high rate of inpatient treatment in Germany and the implications of such a treatment for patients and their families research is definitely required to assess its efficacy in comparison with outpatient treatment. We need to know if this invasive form of treatment has an effect on the proportion of patients with ill health for a period of over 20 years. This proportion has been estimated at 30% [39]. In the context of our study, it would obviously be of interest to assess if reasons for inpatient treatment differ between patients who recover versus those who develop chronic AN.

Our list of reasons (Table 2) obviously does not reflect the complex multifactorial decision-making process for inpatient treatment. A qualitative study addressed at patients, parents and ideally also to referring physicians or psychotherapist, would be useful to obtain an overview of the respective reasons for admission to inpatient treatment. On the basis of such a study, a list of possible reasons for the decision for inpatient treatment could be developed. In addition, a quantitative weighting of such reasons might be useful. Our dichotomous approach entailed a loss of statistical power and did not allow weighting among the reasons.

It should be pointed out that the total number of registered female patients clearly exceeded the 470 for whom both parents and patients provided up to three reasons for seeking inpatient treatment. Nevertheless, descriptive data indicate minimal shifts only in for example age and BMI at admission upon comparison of these 470 patients with the 360 for whom all data used for the purpose of this study were available (Table 1). For reasons of statistical power, we have refrained from separate analyses for the subtypes of AN. Interviews with patients and parents were to be conducted separately. However, using register based data we cannot assure that this was always the case. Future studies could attempt to quantify the relevance of single reasons for admission. As stated above, both age and BMI upon initial body weight loss were recalled by patients or parents; measured height at admission was used as a proxy for height at that time. However, this approach may well underestimate the variance of BMI-SDS at admission explained by premorbid BMI [14].

## Conclusions

Almost 40% of the variance of the BMI at admission for inpatient treatment of pediatric patients with AN can be explained by premorbid BMI, age and the patients or parents stating a low body weight as a major reason for seeking inpatient treatment. Body weight related reasons are frequently endorsed by patients and parents as a reason for inpatient treatment. Somatic symptoms as reasons for seeking treatment are mentioned infrequently only. The concordance of reasons for inpatient treatment given by patients and parents was relatively poor: the highest agreement was found for weight-related items and ‘hyperactivity’. Whereas specific reasons are mostly largely independent of each other, a subgroup of the reasons investigated in the current study clustered particularly consisting of body weight related and somatic items. Further studies that identify the reasons for inpatient treatment, and thus the various symptoms and secondary problems of AN, could help to identify subgroups of patients with AN and thus improve treatment and prognosis.

## Supplementary Information

**Additional file 1: Table S1.** Polychoric correlations between items (reasons for seeking inpatient treatment) given by patients. **Table S2.** Polychoric correlations between items (reasons for seeking inpatient treatment) given by parents. **Table S3. **Results of regression analyses with BMI-SDS as outcome (n = 360) according to model Ib (see Table 4) supplemented by single reasons for inpatient admission based on reasons (total number = 23). **a** Given by patients (each row represents a single regression analysis; slight changes for items premorbid BMI-SDS and age not shown). The change in R2 (ΔR2) indicates the improvement of the model compared to model Ib. Nominally (p < 0.05) significant items/reasons are bolded. **b** Given by parents (each row represents a single regression analysis; slight changes for items premorbid BMI-SDS and age not shown). The change in R2 (ΔR2) indicates the improvement of the model compared to model Ib. Nominally (p < 0.05) significant items/reasons are bolded. **Table S4.** Sample characteristics for post-hoc analyses about BMI-SDS at discharge (n = 337). **Table S5.** Correlation coefficients between measurements at onset of AN, at admission and at discharge. **Table S6.** Results of regression model with BMI-SDS at discharge as outcome (n = 337). **a** According to model in the Additional file 1: Table S6 supplemented by single reasons for inpatient admission based on reasons (total number = 23) given by parents (each row represents a single regression analysis; slight changes for items premorbid BMI-SDS, BMI-SDS at admission, treatment duration, weight gain per treatment week and age not shown). The change in R2 (ΔR2) indicates the improvement of the model compared to model from Additional file 1: Table S4. Nominally (p < 0.05) significant items/reasons are bolded. **b** Including predictors from Additional file 1: Table S6 and single reasons for inpatient admission based on reasons (total number = 23) given by parents. **Figure S1.** Scree plot from principal component analysis of patients data. **Figure S2.** Scree plot from principal component analysis of parents data. **Figure S3.** Histogram of the model residuals from PCA with 4 components, n = 471, patients. **Figure S4.** Histogram of the model residuals from PCA with 4 components, n = 470, parents.

## Data Availability

The datasets analysed during the current study are not publicly available as individual privacy could be compromised, but are available from the corresponding author on reasonable request.

## Abbreviations

AN: Anorexia nervosa
BMI: Body mass index (kg/m^2^)
BMI-SDS: BMI standard deviation score
