# Supplementary material for: Reasons for admission and variance of body weight at referral in female inpatients with anorexia nervosa in Germany
Source: Child Adolesc Psychiatry Ment Health. 2021 Dec 22;15:78. doi: 10.1186/s13034-021-00427-w (PMC8697455; doi:10.1186/s13034-021-00427-w)
Supplement: Supplementary file 1 — Additional file 1: Table S1. Polychoric correlations between items (reasons for seeking inpatient treatment) given by patients. Table S2. Polychoric correlations between items (reasons for seeking inpatient treatment) given by parents. Table S3. Results of regression analyses with BMI-SDS as outcome (n = 360) according to model Ib (see Table 4) supplemented by single reasons for inpatient admission based on reasons (total number = 23). a Given by patients (each row represents a single regression analysis; slight changes for items premorbid BMI-SDS and age not shown). The change in R2 (ΔR2) indicates the improvement of the model compared to model Ib. Nominally (p < 0.05) significant items/reasons are bolded. b Given by parents (each row represents a single regression analysis; slight changes for items premorbid BMI-SDS and age not shown). The change in R2 (ΔR2) indicates the improvement of the model compared to model Ib. Nominally (p < 0.05) significant items/reasons are bolded. Table S4. Sample characteristics for post-hoc analyses about BMI-SDS at discharge (n = 337). Table S5. Correlation coefficients between measurements at onset of AN, at admission and at discharge. Table S6. Results of regression model with BMI-SDS at discharge as outcome (n = 337). a According to model in the Additional file 1: Table S6 supplemented by single reasons for inpatient admission based on reasons (total number = 23) given by parents (each row represents a single regression analysis; slight changes for items premorbid BMI-SDS, BMI-SDS at admission, treatment duration, weight gain per treatment week and age not shown). The change in R2 (ΔR2) indicates the improvement of the model compared to model from Additional file 1: Table S4. Nominally (p < 0.05) significant items/reasons are bolded. b Including predictors from Additional file 1: Table S6 and single reasons for inpatient admission based on reasons (total number = 23) given by parents. Figure S1. Scree plot from principal componen [file 13034_2021_427_MOESM1_ESM.pdf]

Supplementary

Table S1. Polychoric correlations between items (reasons for seeking inpatient treatment) given by patients

Item		a	b	c	d	e	f	g	h	i	j	k	l	m	n	o	p	q	r	s
Low body weight	a	1,00	0,15	**0,38**	-0,21	0,12	**-0,35**	-0,02	-0,08	-0,11	0,08	-0,10	-0,19	-0,26	0,02	-0,16	0,21	0,09	0,15	-0,26
Rapid weight loss	b	0,15	1,00	**0,35**	-0,20	**-0,35**	0,15	-0,13	-0,29	-0,12	0,04	0,07	-0,07	-0,10	**0,49**	0,23	0,12	0,12	-0,08	-0,23
Reduced food intake	c	**0,38**	**0,35**	1,00	**-0,32**	0,02	-0,06	-0,17	-0,12	-0,12	0,01	0,07	-0,18	-0,15	0,02	-0,07	-0,29	**-0,38**	-0,16	**-0,38**
Complete cessation of food and/or fluid intake	d	-0,21	-0,20	**-0,32**	1,00	0,26	**0,37**	0,11	**0,43**	0,09	0,05	0,02	0,21	0,06	0,00	0,18	0,15	0,06	0,14	0,08
Vomiting (self-induced)	e	0,12	**-0,35**	0,02	0,26	1,00	0,05	0,09	0,10	-0,24	0,09	0,06	0,10	0,08	0,02	0,22	0,17	0,09	0,17	0,14
Hyperactivity	f	**-0,35**	0,15	-0,06	**0,37**	0,05	1,00	-0,21	0,01	-0,04	**0,37**	0,14	-0,08	0,17	0,11	**0,30**	0,27	0,18	0,25	0,20
Depressed mood	g	-0,02	-0,13	-0,17	0,11	0,09	-0,21	1,00	0,25	0,17	0,03	-0,20	-0,10	**-0,30**	**-0,34**	-0,18	-0,19	-0,14	-0,22	0,05
Suicidal ideation	h	-0,08	-0,29	-0,12	**0,43**	0,10	0,01	0,25	1,00	-0,16	-0,09	0,02	0,04	0,04	-0,02	0,18	0,13	0,05	0,13	0,10
Social withdrawal	i	-0,11	-0,12	-0,12	0,09	-0,24	-0,04	0,17	-0,16	1,00	-0,16	-0,03	0,18	0,17	-0,21	-0,01	0,13	-0,14	-0,06	0,07
Compulsions	j	0,08	0,04	0,01	0,05	0,09	**0,37**	0,03	-0,09	-0,16	1,00	0,19	**-0,32**	-0,09	-0,01	0,18	**0,35**	0,06	0,14	0,09
Exhaustion	k	-0,10	0,07	0,07	0,02	0,06	0,14	-0,20	0,02	-0,03	0,19	1,00	-0,06	0,02	0,12	**0,33**	0,29	0,19	0,27	0,21
Dizziness	l	-0,19	-0,07	-0,18	0,21	0,10	-0,08	-0,10	0,04	0,18	**-0,32**	-0,06	1,00	**0,45**	0,07	-0,05	0,11	-0,01	-0,09	**0,36**
Low heart rate	m	-0,26	-0,10	-0,15	0,06	0,08	0,17	**-0,30**	0,04	0,17	-0,09	0,02	**0,45**	1,00	0,00	**0,37**	0,13	0,24	0,12	**0,59**
Low blood pressure	n	0,02	**0,49**	0,02	0,00	0,02	0,11	**-0,34**	-0,02	-0,21	-0,01	0,12	0,07	0,00	1,00	**0,30**	0,27	0,17	0,24	0,17
Abnormal electrocardiogram	o	-0,16	0,23	-0,07	0,18	0,22	**0,30**	-0,18	0,18	-0,01	0,18	**0,33**	-0,05	**0,37**	**0,30**	1,00	**0,51**	**0,37**	**0,45**	**0,38**
Abnormal blood laboratory values	p	0,21	0,12	-0,29	0,15	0,17	0,27	-0,19	0,13	0,13	**0,35**	0,29	0,11	0,13	0,27	**0,51**	1,00	**0,34**	**0,41**	**0,32**
Increase in school absenteeism	q	0,09	0,12	**-0,38**	0,06	0,09	0,18	-0,14	0,05	-0,14	0,06	0,19	-0,01	0,24	0,17	**0,37**	**0,34**	1,00	**0,31**	**0,44**
Family conflicts	r	0,15	-0,08	-0,16	0,14	0,17	0,25	-0,22	0,13	-0,06	0,14	0,27	-0,09	0,12	0,24	**0,45**	**0,41**	**0,31**	1,00	**0,33**
Recommendation of physician or therapist	s	-0,26	-0,23	**-0,38**	0,08	0,14	0,20	0,05	0,10	0,07	0,09	0,21	**0,36**	**0,59**	0,17	**0,38**	**0,32**	**0,44**	**0,33**	1,00

correlation coefficients ≥ 0.30 are marked red

Table S2. Polychoric correlations between items (reasons for seeking inpatient treatment) given by parents

Item		a	b	c	d	e	f	g	h	i	j	l	m	n	o	p	q	s	t	u
Low body weight	a	1,00	-0,28	0,19	-0,22	-0,24	-0,10	-0,16	**-0,34**	-0,09	-0,06	-0,06	0,12	0,11	-0,05	0,00	-0,03	**-0,55**	-0,08	0,21
Rapid weight loss	b	-0,28	1,00	0,18	-0,07	-0,01	-0,21	-0,11	-0,13	-0,15	-0,12	-0,30	-0,12	-0,09	-0,24	0,08	-0,15	-0,05	-0,21	0,02
Reduced food intake	c	0,19	0,18	1,00	**-0,41**	-0,05	-0,24	-0,20	-0,17	-0,07	**-0,32**	-0,03	-0,08	**-0,30**	-0,28	-0,20	**-0,38**	**-0,30**	-0,17	-0,12
Complete cessation of food and/or fluid intake	d	-0,22	-0,07	**-0,41**	1,00	-0,09	0,11	-0,08	0,10	-0,20	0,28	0,11	-0,04	-0,13	0,11	0,21	0,15	0,09	0,32	0,08
Vomiting (self-induced)	e	-0,24	-0,01	-0,05	-0,09	1,00	0,02	0,17	0,18	0,04	-0,07	0,03	0,02	-0,06	0,17	0,11	0,22	0,16	-0,17	-0,25
Hyperactivity	f	-0,10	-0,21	-0,24	0,11	0,02	1,00	0,02	0,07	0,11	-0,02	-0,07	0,07	-0,01	0,23	0,16	0,28	0,22	0,27	**-0,32**
Depressed mood	g	-0,16	-0,11	-0,20	-0,08	0,17	0,02	1,00	0,10	0,13	0,08	0,10	-0,22	-0,11	-0,05	-0,26	0,02	**0,30**	-0,08	-0,29
Suicidal ideation	h	**-0,34**	-0,13	-0,17	0,10	0,18	0,07	0,10	1,00	-0,07	-0,03	0,08	0,06	0,14	0,23	0,16	0,29	0,22	-0,12	-0,08
Social withdrawal	i	-0,09	-0,15	-0,07	-0,20	0,04	0,11	0,13	-0,07	1,00	0,08	-0,22	-0,19	-0,01	-0,04	0,05	0,01	0,13	0,14	**-0,39**
Compulsions	j	-0,06	-0,12	**-0,32**	0,28	-0,07	-0,02	0,08	-0,03	0,08	1,00	0,14	-0,02	0,04	0,13	0,07	0,17	0,11	0,11	**-0,30**
Exhaustion	l	-0,06	**-0,30**	-0,03	0,11	0,03	-0,07	0,10	0,08	-0,22	0,14	1,00	-0,06	0,16	0,29	0,02	0,14	0,08	-0,27	-0,02
Dizziness	m	0,12	-0,12	-0,08	-0,04	0,02	0,07	-0,22	0,06	-0,19	-0,02	-0,06	1,00	-0,01	**0,42**	0,16	0,26	**0,40**	-0,23	0,23
Low heart rate	n	0,11	-0,09	**-0,30**	-0,13	-0,06	-0,01	-0,11	0,14	-0,01	0,04	0,16	-0,01	1,00	**0,34**	0,08	0,20	0,13	-0,07	0,08
Low blood pressure	o	-0,05	-0,24	-0,28	0,11	0,17	0,23	-0,05	0,23	-0,04	0,13	0,29	**0,42**	**0,34**	1,00	**0,31**	**0,45**	**0,38**	-0,09	-0,19
Abnormal electrocardiogram	p	0,00	0,08	-0,20	0,21	0,11	0,16	-0,26	0,16	0,05	0,07	0,02	0,16	0,08	**0,31**	1,00	**0,60**	**0,30**	0,00	-0,10
Abnormal blood laboratory values	q	-0,03	-0,15	**-0,38**	0,15	0,22	0,28	0,02	0,29	0,01	0,17	0,14	0,26	0,20	**0,45**	**0,60**	1,00	**0,46**	-0,04	0,06
Increase in school absenteeism	s	**-0,55**	-0,05	**-0,30**	0,09	0,16	0,22	**0,30**	0,22	0,13	0,11	0,08	**0,40**	0,13	**0,38**	**0,30**	**0,46**	1,00	-0,10	-0,04
Family conflicts	t	-0,08	-0,21	-0,17	**0,32**	-0,17	0,27	-0,08	-0,12	0,14	0,11	-0,27	-0,23	-0,07	-0,09	0,00	-0,04	-0,10	1,00	-0,06
Recommendation of physician or therapist	u	0,21	0,02	-0,12	0,08	-0,25	**-0,32**	-0,29	-0,08	**-0,39**	-0,30	-0,02	0,23	0,08	-0,19	-0,10	0,06	-0,04	-0,06	1,00

correlation coefficients ≥ 0.30 are marked red

Figure S1. Scree plot from principal component analysis of patients data

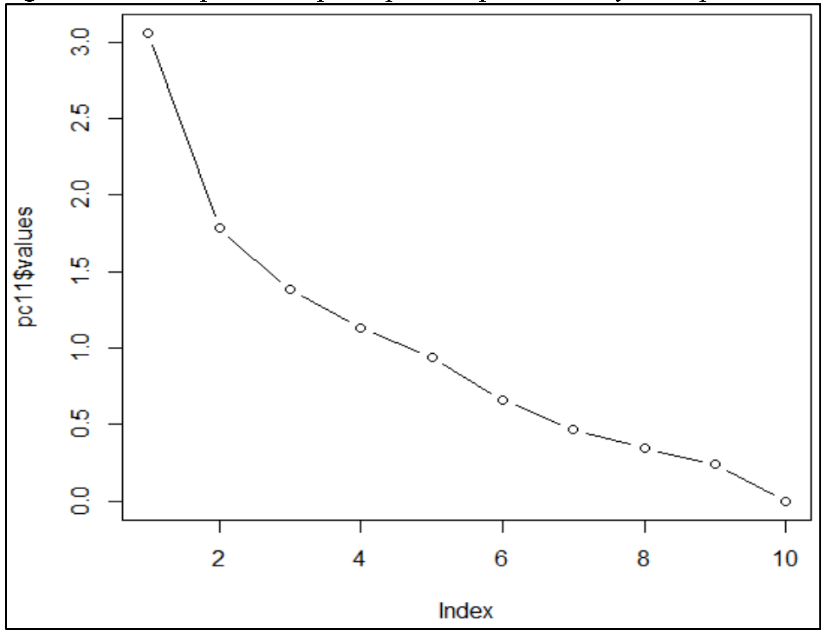

Figure S2. Scree plot from principal component analysis of parents data

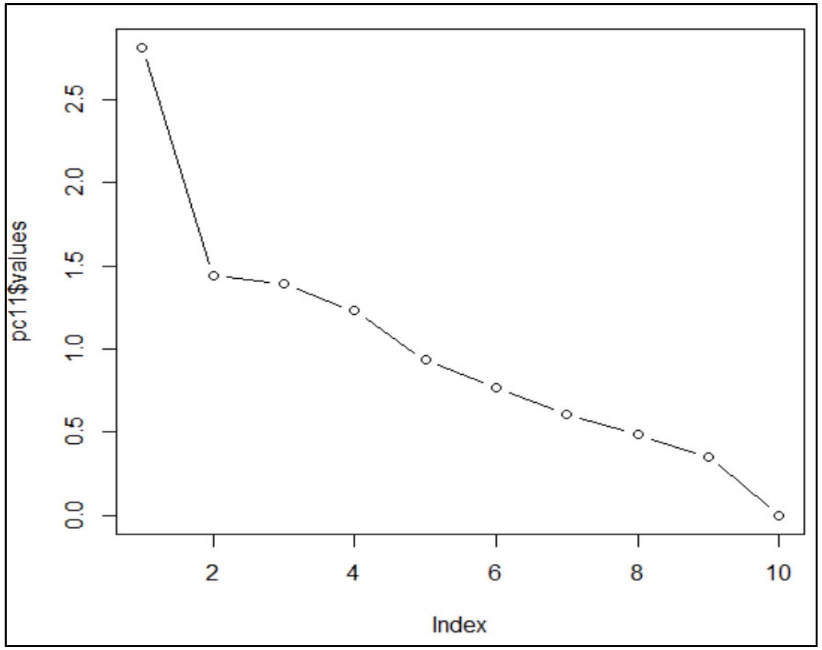

Figure S3. Histogram of the model residuals from PCA with 4 components, n=471, patients

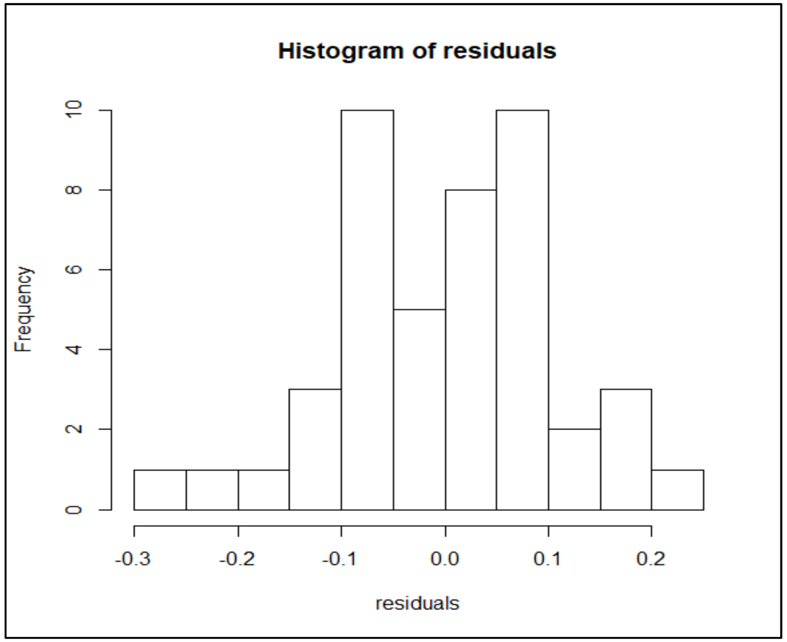

Figure S4. Histogram of the model residuals from PCA with 4 components, n=470, parents

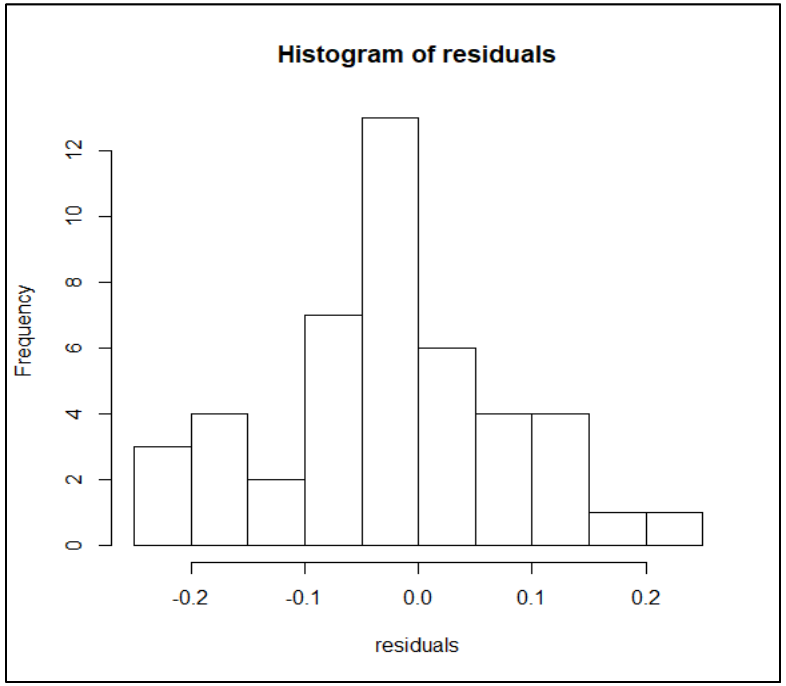

Table S3a: Results of regression analyses with BMI-SDS as outcome (n=360) according to model Ib (see Table 4) supplemented by single reasons for inpatient admission based on reasons (total number = 23) given by *patients* (each row represents a single regression analysis; slight changes for items premorbid BMI-SDS and age not shown). The change in R^2 (ΔR^2) indicates the improvement of the model compared to model Ib. Nominally ($p < 0.05$) significant items/reasons are bolded.

Item	B	SE	Beta	T	Sig	ΔR^2 (%)
Low body weight	-0.45	0.10	-0.19	-4.43	**1.25 x 10^{-05}**	3.49
Rapid weight loss	-0.11	0.11	-0.04	-0.99	0.320	0.19
Reduced food intake	-0.10	0.11	-0.04	-0.95	0.341	0.17
Complete cessation of food and/or fluid intake	7.88 x10^{-05}	0.23	0	0	1.000	0.00
Vomiting (self-induced)	0.32	0.29	0.05	1.10	0.272	0.23
Hyperactivity	0.29	0.31	0.04	0.93	0.353	0.16
Depressed mood	0.31	0.12	0.11	2.61	**0.009**	1.26
Suicidal ideation	0.41	0.23	0.08	1.79	0.073	0.60
Social withdrawal	0.02	0.15	0.004	0.1	0.920	0.00
Compulsions	0.06	0.25	0.01	0.25	0.807	0.01
Other mental problems	-0.10	0.37	-0.01	-0.27	0.787	0.00
Exhaustion	-0.04	0.14	-0.01	-0.28	0.781	0.01
Dizziness	0.26	0.26	0.04	1.01	0.315	0.19
Low heart rate	-0.34	0.28	-0.05	-1.20	0.231	0.27
Low blood pressure	-0.59	0.68	-0.04	-0.87	0.386	0.14
Abnormal electrocardiogram	-0.39	0.49	-0.04	-0.80	0.422	0.12
Abnormal blood laboratory values	0.07	0.44	0.01	0.16	0.871	0.00
Other somatic symptoms	-0.18	0.49	-0.02	-0.36	0.716	0.02
Increase in school absenteeism	0.35	0.49	0.03	0.73	0.469	0.10
Family conflicts	0.04	0.15	0.01	0.26	0.798	0.01
Recommendation of physician or therapist	-0.01	0.11	-0.004	-0.09	0.923	0.00
No reason for admission	-0.33	0.21	-0.07	-1.58	0.116	0.46
Other reasons	0.55	0.17	0.14	3.19	**0.002**	1.86

B: Unstandardized regression coefficients

SE: Standard error of regression coefficients

Beta: Standardized regression coefficients

ΔR^2: Change of the explained variance by the model compared to model Ib (by percent)

Table S3b: Results of regression analyses with BMI-SDS as outcome (n=360) according to model Ib (see Table 4) supplemented by single reasons for inpatient admission based on reasons (total number = 23) given by *parents* (each row represents a single regression analysis; slight changes for items premorbid BMI-SDS and age not shown). The change in R^2 (ΔR^2) indicates the improvement of the model compared to model Ib. Nominally ($p < 0.05$) significant items/reasons are bolded.

Item	B	SE	Beta	T	Sig	ΔR^2 (%)
Low body weight	-0.57	0.11	-0.23	-5.44	**1.00 x 10^{-07}**	5.12
Rapid weight loss	-0.03	0.10	-0.01	-0.32	0.749	0.02
Reduced food intake	-0.14	0.10	-0.06	-1.33	0.184	0.33
Complete cessation of food and/or fluid intake	0.11	0.25	0.02	0.44	0.660	0.04
Vomiting (self-induced)	0.76	0.25	0.13	3.00	**0.003**	1.65
Hyperactivity	0.11	0.28	0.02	0.39	0.700	0.03
Depressed mood	0.31	0.12	0.12	2.69	**0.007**	1.34
Suicidal ideation	0.80	0.31	0.11	2.61	**0.010**	1.25
Social withdrawal	0.41	0.16	0.11	2.53	**0.012**	1.18
Compulsions	0.20	0.25	0.04	0.81	0.418	0.12
Other mental problems	-0.17	0.49	-0.02	-0.35	0.725	0.02
Exhaustion	0.06	0.22	0.01	0.26	0.799	0.01
Dizziness	-0.16	0.35	-0.02	-0.46	0.646	0.04
Low heart rate	-0.15	0.24	-0.03	-0.65	0.519	0.08
Low blood pressure	-0.45	0.56	-0.04	-0.79	0.425	0.12
Abnormal electrocardiogram	-0.81	0.39	-0.09	-2.04	**0.042**	0.77
Abnormal blood laboratory values	-0.94	0.56	-0.07	-1.68	0.094	0.52
Other somatic symptoms	-0.26	0.69	-0.02	-0.37	0.711	0.03
Increase in school absenteeism	0.45	0.49	0.04	0.92	0.358	0.16
Family conflicts	0.16	0.15	0.05	1.07	0.287	0.21
Recommendation of physician or therapist	-0.27	0.12	-0.09	-2.25	**0.025**	0.94
No reason for admission	0.17	0.97	0.01	0.18	0.859	0.01
Other reasons	0.28	0.21	0.06	1.34	0.182	0.33

B: Unstandardized regression coefficients

SE: Standard error of regression coefficients

Beta: Standardized regression coefficients

ΔR^2: Change of the explained variance by the model compared to model Ib (by percent)

Post-hoc analyses on the association of BMI-SDS at discharge and reasons for admission

Table S4. Sample characteristics for post-hoc analyses about BMI-SDS at discharge (n=337)

	Mean	SD
Treatment duration (weeks)	17.21	8.47
Weight gain (kg) per treatment	8.19	4.23
Weight gain (kg) per treatment week	0.58	0.65
BMI at discharge	18.26	1.43
BMI-SDS at discharge	-1.15	0.77

SD: Standard deviation; BMI: body mass index; BMI-SDS: BMI standard deviation score

Table S5. Correlation coefficients between measurements at onset of AN, at admission and at discharge

		BMI-SDS at discharge	BMI-SDS premorbid	BMI-SDS at admission	Treatment duration	Weight gain (kg) per treatment week
BMI-SDS at discharge	r	1	.332	.462	.078	.057
	p		4.37×10^{-10}	3.09×10^{-19}	0.153	0.295
BMI-SDS premorbid	r		1	.472	-.106	-.089
	p			4.14×10^{-20}	0.051	0.103
BMI-SDS at admission	r			1	-.212	-.148
	p				8.50×10^{-05}	0.006
Treatment duration	r				1	-.340
	p					1.39×10^{-10}

r: correlation coefficient; p: significance

Table S6. Results of regression model with BMI-SDS at discharge as outcome (n=337)

Predictors	B	SE	Beta	T	Sig.
Intercept	0.451	0.345		1.307	0.192
BMI-SDS premorbid	0.161	0.044	0.189	3.666	**2.87×10^{-04}**
BMI-SDS at admission	0.263	0.037	0.405	7.136	**6.09×10^{-12}**
Age at admission	-0.090	0.024	-0.183	-3.783	**1.84×10^{-04}**
Treatment duration	0.024	0.004	0.269	5.427	**1.11×10^{-07}**
Weight gain (kg) per treatment week	0.287	0.058	0.242	4.928	**1.31×10^{-06}**

Model R^2=0.34

B: Unstandardized regression coefficients; SE: Standard error of unstandardized regression coefficient; Beta: Standardized coefficients; t: t-statistic; Sig: significance; R^2: explained variance

Table S6a. Results of regression analyses with BMI-SDS at discharge as outcome (n=337) according to model in the Table S6 supplemented by single reasons for inpatient admission based on reasons (total number = 23) given by **parents** (each row represents a single regression analysis; slight changes for items premorbid BMI-SDS, BMI-SDS at admission, treatment duration, weight gain per treatment week and age not shown). The change in R^2 (ΔR^2) indicates the improvement of the model compared to model from Table S4. Nominally ($p < 0.05$) significant items/reasons are bolded.

Predictors	B	SE	Beta	T	Sig.	ΔR^2 (%)
Low body weight	-0.017	0.076	-0.01	-0.218	0.828	0.01
Rapid weight loss	-0.100	0.070	-0.065	-1.422	0.156	0.40
Reduced food intake	0.117	0.069	0.076	1.695	0.091	0.57
Complete cessation of food and/or fluid intake	-0.156	0.168	-0.042	-0.929	0.354	0.17
Vomiting (self-induced)	0.028	0.183	0.007	0.151	0.880	0.00
Hyperactivity	-0.160	0.194	-0.037	-0.823	0.411	0.14
Depressed mood	-0.063	0.081	-0.035	-0.779	0.437	0.12
Suicidal ideation	0.099	0.206	0.022	0.480	0.632	0.05
Social withdrawal	0.066	0.110	0.027	0.604	0.546	0.07
Compulsions	-0.033	0.164	-0.009	-0.200	0.841	0.01
Other mental problems	**-0.911**	**0.314**	**-0.129**	**-2.904**	**0.004**	**1.65**
Exhaustion	0.107	0.142	0.034	0.754	0.451	0.11
Dizziness	0.185	0.241	0.034	0.767	0.443	0.12
Low heart rate	-0.118	0.168	-0.032	-0.698	0.485	0.10
Low blood pressure	-0.197	0.367	-0.024	-0.536	0.593	0.06
Abnormal electrocardiogram	0.021	0.263	0.004	0.080	0.936	0.00
Abnormal blood laboratory values	0.072	0.369	0.009	0.195	0.845	0.01
Other somatic symptoms	0.009	0.449	0.001	0.020	0.984	0.00
Increase in school absenteeism	0.195	0.318	0.028	0.612	0.541	0.08
Family conflicts	-0.039	0.099	-0.018	-0.395	0.693	0.03
Recommendation of physician or therapist	**0.262**	**0.079**	**0.149**	**3.328**	**0.001**	2.15
No reason for admission	0.200	0.633	0.014	0.317	0.752	0.02
Other reasons	0.120	0.148	0.037	0.811	0.418	0.13

Model R^2=0.41

B: Unstandardized regression coefficients; SE: Standard error of unstandardized regression coefficient; Beta: Standardized coefficients; t: t-statistic; Sig: significance; R^2: explained variance

Table S6b. Results of regression analyses with BMI-SDS at discharge as outcome (n=337) including predictors from Table S6 and single reasons for inpatient admission based on reasons (total number = 23) given by **parents**.

Predictors*	B	SE	Beta	T	Sig.**
Intercept	0.292	0.340		0.858	0.392
BMI-SDS premorbid	0.158	0.043	0.186	3.693	**2.60×10^{-04}**
BMI-SDS at admission	0.275	0.036	0.423	7.594	**3.25×10^{-13}**
Age at admission	-0.081	0.023	-0.165	-3.465	**6.00×10^{-04}**
Treatment duration	0.025	0.004	0.278	5.740	**2.16×10^{-08}**
Weight gain (kg) per treatment week	0.272	0.057	0.229	4.769	**2.79×10^{-06}**
Other mental problems	-0.847	0.310	-0.120	-2.730	**7.31×10^{-03}**
Recommendation of physician or therapist	0.248	0.078	0.141	3.175	**1.64×10^{-03}**

Model R^2=0.37

*all items rendering nominal P-values <0.05 based on reasons for inpatient treatment given by parents

**predictors that survive the Bonferroni-Holm correction are highlighted (9 tests in model for parents)

B: Unstandardized regression coefficients; SE: Standard error of unstandardized regression coefficient; Beta: Standardized coefficients; t: t-statistic; Sig: significance; R^2: explained variance
